# Systems approach to integrative oncology in breast cancer care: service design, delivery and patient experiences from a UK center

**DOI:** 10.3389/fonc.2026.1870158

**Published:** 2026-06-29

**Authors:** Nina Fuller-Shavel, Lauren Watts, Nazanin Derakshan

**Affiliations:** 1 Department of Integrative Cancer Care, Synthesis Clinic, Reading, Berkshire, United Kingdom; 2 National Centre for Integrative Oncology (NCIO), Reading, Berkshire, United Kingdom

**Keywords:** breast cancer, cancer nutrition, cancer rehabilitation, cancer survivorship, integrative oncology, mistletoe therapy, psycho-oncology, supportive care

## Abstract

**Background:**

Breast cancer affects 2.3 million women annually worldwide, with survivors experiencing substantial unmet supportive care needs around symptom management, lifestyle support, psychological wellbeing, sexual health and survivorship. Integrative oncology (IO) is a patient-centered, evidence-informed approach combining lifestyle and mind-body interventions, natural products and complementary therapies alongside standard cancer treatment, supported by Society for Integrative Oncology and American Society of Clinical Oncology (SIO-ASCO) guidelines, but UK service delivery and patient experience data are limited.

**Methods:**

We conducted a multi-component study combining (i) a four-year electronic healthcare record (EHR) analysis of breast cancer (BC) patients attending Synthesis Clinic, a UK physician-led multidisciplinary integrative oncology service, between January 2022 and March 2026; and (ii) a cross-sectional patient survey of 232 women diagnosed with breast cancer, in collaboration with the National Centre for Integrative Oncology (NCIO) and the UK Building Resilience in Breast Cancer (BRiC) Centre, capturing experience of standard oncological care alone (n=211) and of combined standard plus integrative oncology care (n=21).

**Results:**

EHR analysis identified 182 women (mean age 50.3 years; 45.6% ever-metastatic; 20.3% triple-negative). Modality engagement was anchored on nutrition (95.0%) and integrative oncology physician consultations (55.9%), with smaller subgroups accessing intravenous nutrient therapy (20.9%), mistletoe therapy (13.7%), emotional wellbeing support (13.2%) and other modalities. In the standard care survey, unmet need exceeded 60% across seven supportive care domains, peaking at 85% for sleep and 83% for sexual health. Within-patient comparison in the IO cohort showed unmet need reduced to 0% in 11 of 13 directly comparable domains, with reductions exceeding 50 percentage points in 8 domains. 76% of IO cohort respondents rated integrative care as extremely important and 88% rated quality as very high or high.

**Conclusions:**

A structured, multidisciplinary, physician-led integrative oncology service is feasible in the UK and may help address guideline-aligned supportive care domains that are systematically under-delivered within standard oncological care. Substantial sociodemographic inequities in access remain and are the explicit focus of the NCIO charity service development. Our findings may also support the case for developing multidisciplinary integrative oncology hubs as specialist community centers within the NHS.

## 1 Introduction

Breast cancer is the most commonly diagnosed cancer worldwide, with an estimated 2.3 million new cases annually. It accounts for around 11.7% of all cancer diagnoses (1). Breast cancer remains a leading cause of cancer-related death among women, with over 680,000 deaths reported globally each year (1). In the United Kingdom, breast cancer represents around 15% of all new cancer cases, with incidence rates continuing to rise due to population ageing and improved detection (2). Advances in earlier detection and systemic therapies have significantly improved survival, and 5-year survival now exceeds 85% in high-income countries (3, 4). However, these gains are accompanied by a growing population of survivors experiencing long-term treatment-related morbidity.

Breast cancer is a biologically heterogeneous disease comprising distinct molecular subtypes with differing prognoses and therapeutic implications. These include hormone receptor HR-positive and HER2-negative disease, which accounts for approximately 70% of cases and is generally associated with a favorable prognosis; HER2-positive breast cancer, accounting for 15–20%, characterized by overexpression of the HER2 receptor and improved outcomes due to targeted therapies; and triple-negative breast cancer (TNBC) accounting for 10–15%, defined by the absence of estrogen receptor (ER), progesterone receptor (PR), and HER2 expression. This is associated with more aggressive clinical behavior and fewer targeted treatment options (5, 6). These subtypes differ not only in oncological outcomes but also in treatment-related toxicity profiles and survivorship challenges, reinforcing the need for personalized supportive care strategies.

Despite therapeutic advances, patients with breast cancer commonly experience significant symptom burden during and after treatment. Fatigue affects up to 80% of patients undergoing chemotherapy, while anxiety, depression, pain, sleep disturbance, and chemotherapy-induced nausea and vomiting (CINV) are also commonly reported, with a high prevalence of unmet support needs (7–10). Long-term endocrine therapy with aromatase inhibitors, such as letrozole, anastrozole and exemestane, is associated with arthralgia, vasomotor symptoms, and reduced quality of life, often contributing to suboptimal adherence (11, 12). These factors highlight the limitations of the current delivery of supportive care and the need for comprehensive approaches that address physical and psychosocial outcomes.

Integrative oncology has emerged as an evidence-informed, patient-centered approach that combines lifestyle modifications, such as physical activity and nutritional strategies, natural products, mind-body interventions, and complementary therapies, such as acupuncture, to support people undergoing standard cancer treatment (13). Importantly, integrative oncology is focused on safe and effective support and coordination with conventional care rather than alternative or unproven treatments (13, 14). Its relevance in breast cancer is underscored by high utilization rates. Up to 37–82% of breast cancer patients, depending on location, care setting and definitions used, report using some form of complementary therapy along their cancer treatment pathway with the average in Western Europe being approximately 50% (15–20).

The clinical integration of evidence-based integrative approaches has been supported by the American Society of Clinical Oncology (ASCO) and the Society for Integrative Oncology (SIO), which jointly developed guidelines on symptom control, including depression, anxiety, pain and fatigue (7, 21, 22), with prior endorsement of the SIO guideline on breast cancer support (23). Guidelines on integrative support in breast cancer synthesized data from randomized controlled trials and provided graded recommendations for specific interventions, although recommendations need to be updated, given the burgeoning amount of new data since the guideline publication in 2017 (24). In the published breast cancer guidelines, there is strong evidence supporting the use of meditation, yoga, and stress management for reducing anxiety, depression, and stress (Grade A recommendations), while acupuncture and acupressure are recommended for managing CINV (Grade B) (23, 24). Additional modalities, such as mindfulness-based interventions and physical activity, have demonstrated benefit in improving quality of life and fatigue. Conversely, the guidelines advise against routine use of many dietary supplements, emphasizing the importance of clinician oversight.

The need for integrative oncology in breast cancer care is driven by persistent gaps in symptom management, survivorship care, and patient experience (10, 25). Uncontrolled symptoms negatively impact treatment adherence, clinical outcomes, and healthcare utilization (25). Furthermore, many patients independently seek complementary therapies, often without guidance, or without disclosing to their conventional medical team, increasing the risk of interactions or ineffective use. Integrative oncology (IO) provides a structured, evidence-based framework to address these issues, aligning patient preferences with safe and effective supportive care, provided that IO care is delivered with a multidisciplinary medically led or supervised framework.

Cancer is increasingly understood as a chronic, non-linear, multi-system condition in which biological, psychological, behavioral and social processes interact across the disease trajectory and beyond active treatment. At the biological level, contemporary expansions of the hallmarks of cancer framework now incorporate polymorphic microbiomes, non-mutational epigenetic reprogramming, phenotypic plasticity and senescent cells as enabling characteristics alongside the classical hallmarks, and explicitly conceptualize cancer as a systemic disease shaped by host metabolic, immune, circadian, neural and microbial environments (26–29). In parallel, systems medicine has identified several interconnected biological hubs, including oxidative stress, chronic inflammation, extracellular matrix remodeling, metabolic dysregulation and immune signaling, that act as integrative nodes underpinning the chronicity of non-communicable disease (30, 31). At the level of care delivery, healthcare is now widely characterized as a complex adaptive system in which fuzzy boundaries, non-linearity and emergent behavior make purely reductionist single-modality approaches insufficient for chronic conditions (32), with refreshed conceptual frameworks for supportive and survivorship care similarly calling for reconfiguration as a continuous, multidisciplinary platform delivering a person-centered approach (33, 34). The work described here is explicitly positioned within a systems-oriented approach, relevant to both cancer pathophysiology and care delivery, and this is operationalized through the Systems Approach to Cancer Framework (SACF) introduced in Methods.

Synthesis Clinic is a physician-led multidisciplinary integrative oncology practice in the United Kingdom. The clinic’s approach combines input from IO physicians, nurses and pharmacy team with nutrition, lifestyle medicine, health coaching, psycho-oncology, cancer rehabilitation and other multimodal input, including but not limited to mistletoe therapy, herbal medicine, acupuncture and yoga therapy, within a multidisciplinary team (MDT), working alongside primary care and oncology providers. This specialist clinic operates as a Care Quality Commission (CQC)-registered outpatient service with a dedicated leadership and operations team supporting clinical, pharmacy and administrative functions, including a Medical Director, Practice Manager, Pharmacy Lead and Nursing Lead. Governance is anchored in standardized operating procedures, an integrated electronic health record (EHR), structured intake and consent workflows, and an embedded clinical audit and outcomes program that uses a defined panel of patient-reported outcome measures (PROMIS Global Health, MDASI, MSQ and WEMWBS as a core set, with additional scores delivered as needed). Medication, supplement and herbal medicine safety is overseen by an integrative clinical pharmacist, with all supplement and herbal prescriptions systematically screened for drug-supplement and herb-drug interactions by both clinicians and pharmacy staff, and additional safety monitoring (including regular hematology and biochemistry surveillance) applied to specific modalities, such as mistletoe (*Viscum album* extract) therapy as previously described (35, 36).

**Figure 1:**
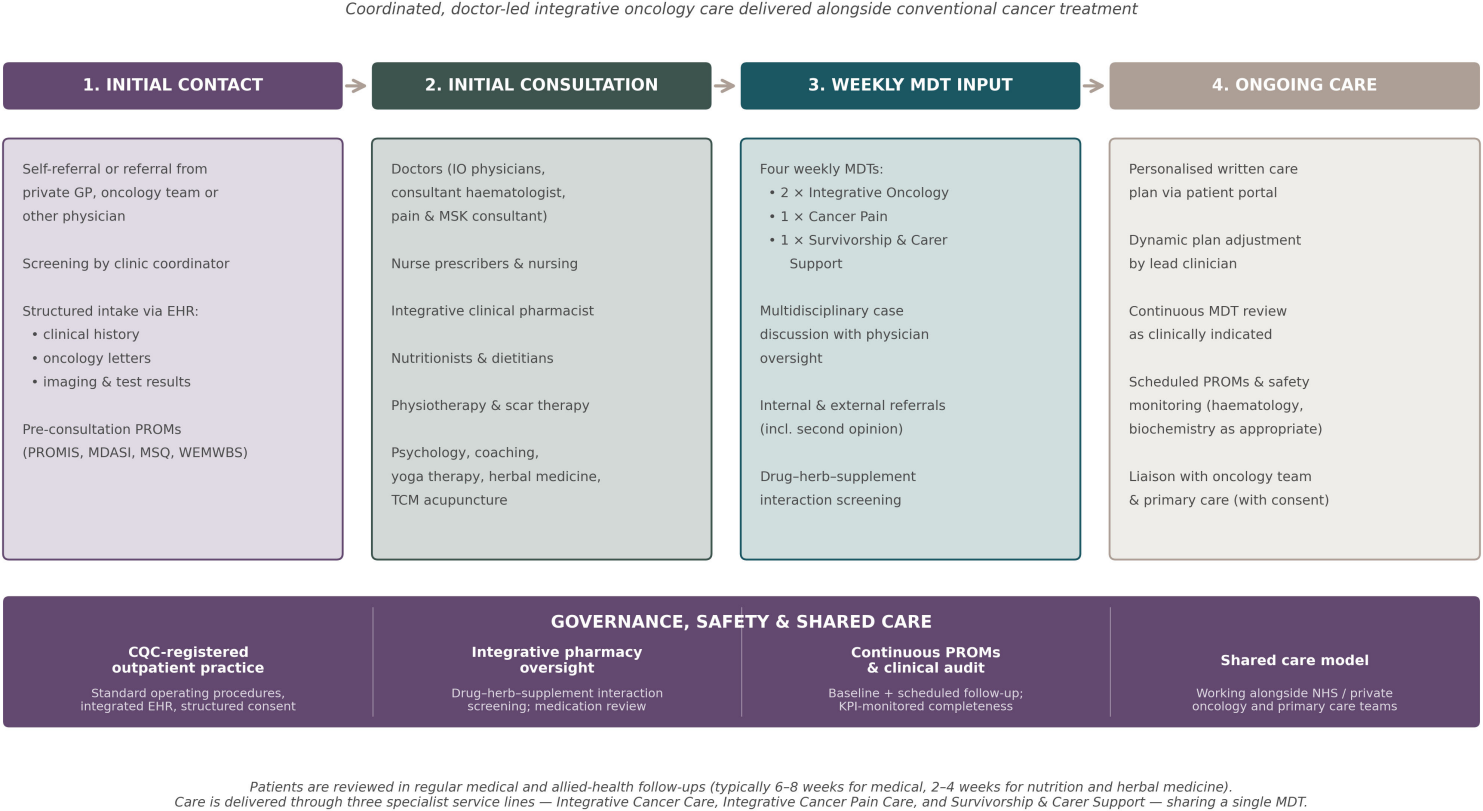
Synthesis Clinic patient pathway and multidisciplinary team process. EHR, electronic health record; KPI, key performance indicator; MDASI, M.D. Anderson Symptom Inventory; MDT, multidisciplinary team; MSK, musculoskeletal; MSQ, Medical Symptoms Questionnaire; PROMs, patient-reported outcome measures; PROMIS, Patient-Reported Outcomes Measurement Information System; TCM, Traditional Chinese Medicine; WEMWBS, Warwick-Edinburgh Mental Wellbeing Scale.

**Figure 2:**
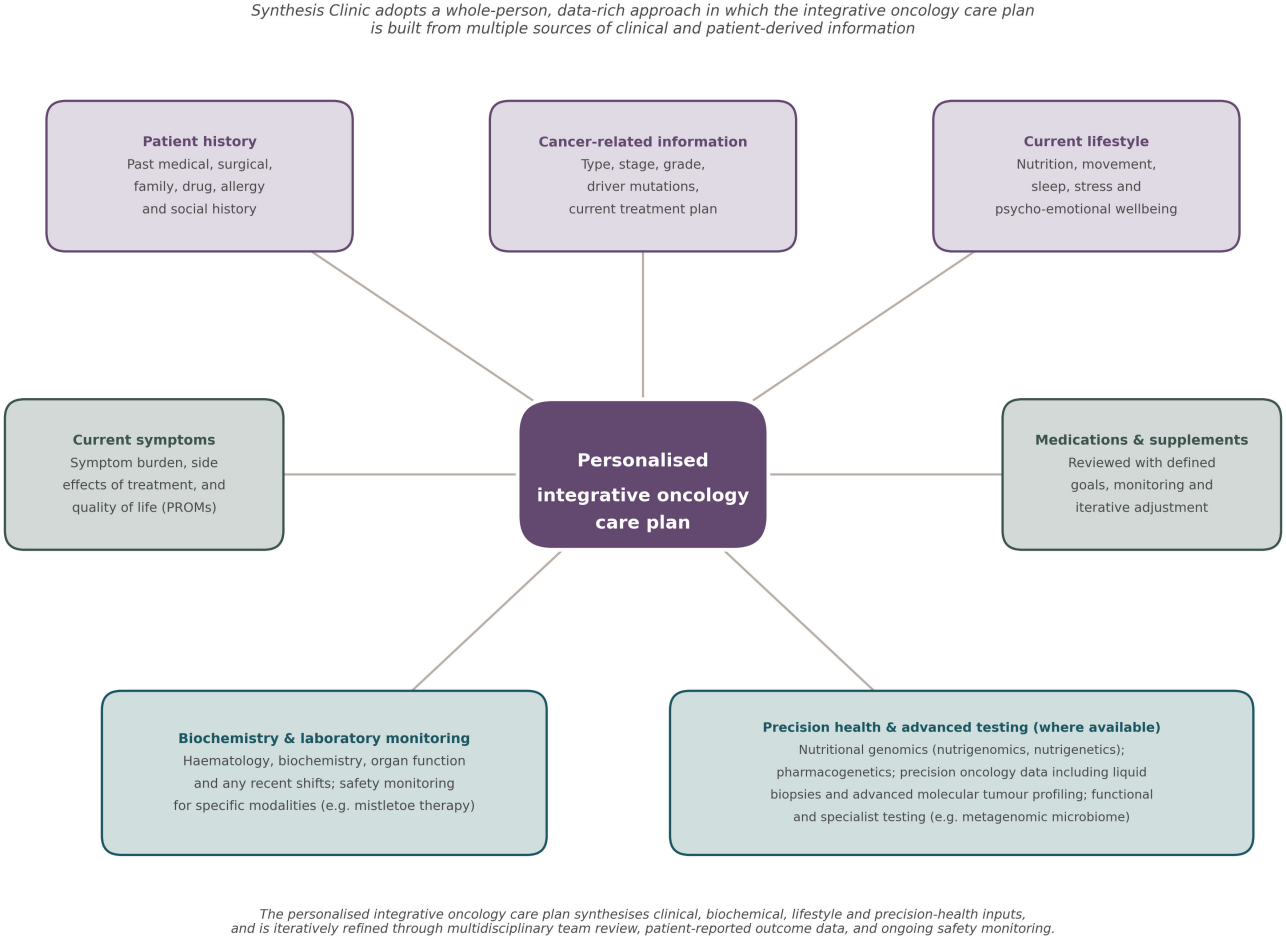
A summary of information inputs that integrative oncology physicians at Synthesis Clinic use to guide multimodal personalized care plans. Multi-source information is synthesized and aligned to patient needs, goals and preferences to produce a dynamically evolving integrative oncology care plan. MDT, multidisciplinary team; PROMs, patient-reported outcome measures.

**Figure 3:**
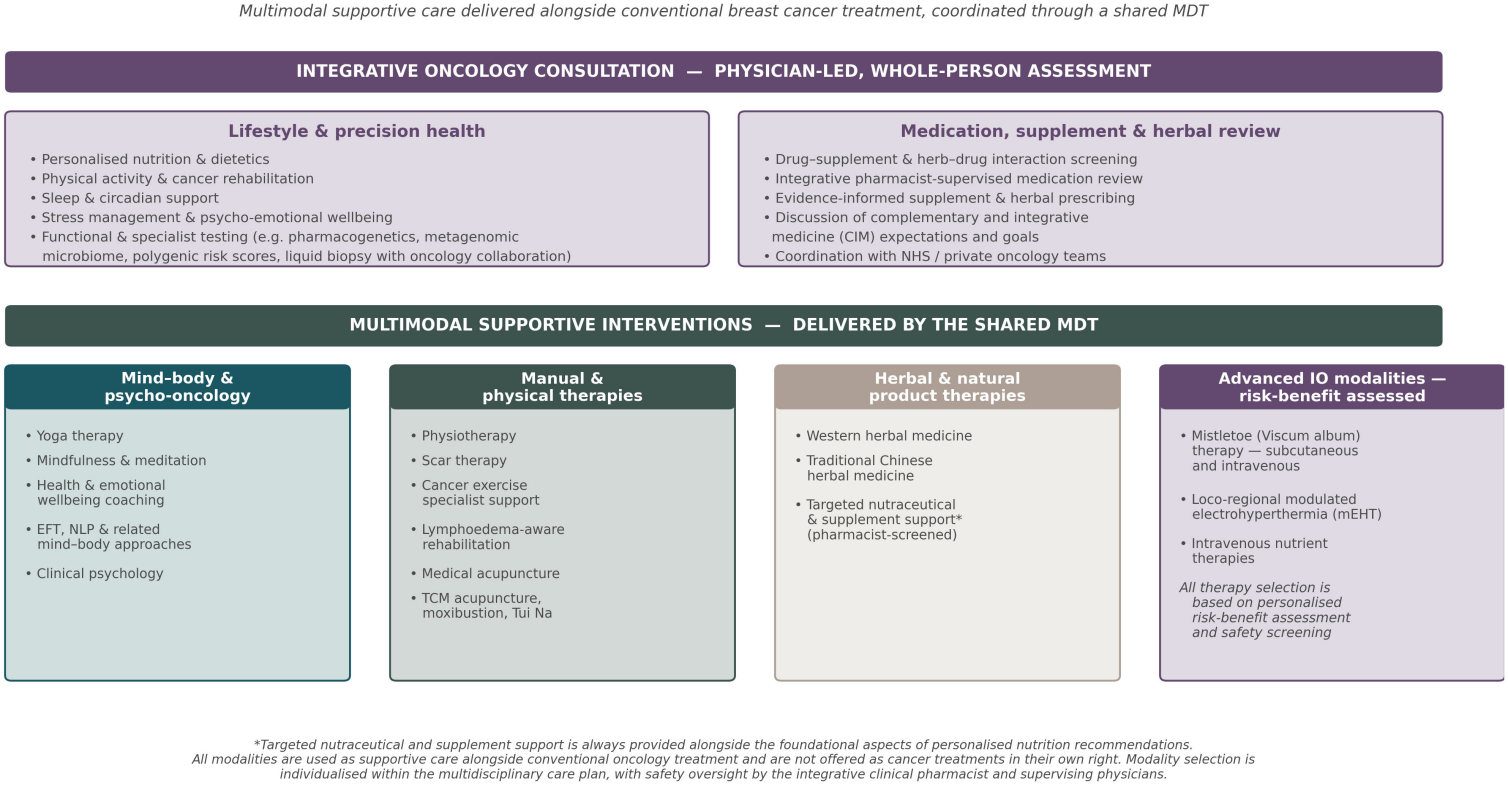
Multimodal supportive and integrative care delivered at Synthesis Clinic alongside conventional breast cancer treatment. CIM, complementary and integrative medicine; EFT, Emotional Freedom Techniques; IO, integrative oncology; MDT, multidisciplinary team; mEHT, modulated electrohyperthermia; NLP, neuro-linguistic programming; TCM, Traditional Chinese Medicine.

Supportive and integrative breast cancer care at Synthesis Clinic is delivered by a diverse healthcare professional team working across three specialist departments (Integrative Cancer Care, Integrative Cancer Pain Care, and Survivorship & Carer Support) and coordinated through four MDT (multidisciplinary team) meetings per week, which cover structured case discussion, internal and external referrals, and continuity of physician oversight throughout the patient journey. The MDT brings together integrative oncology and other specialist physicians, such as consultants in hematology and pain medicine, registered nurses and nurse prescribers, BANT-registered nutritional therapy practitioners and dietitians, physiotherapists with cancer rehabilitation and scar therapy expertise, as well as clinical psychology and emotional wellbeing coaching, yoga therapy, Western and Chinese herbal medicine and licensed acupuncturist input. This structure enables coordinated assessment using the Systems Approach to Cancer Framework followed by delivery of a personalized care plan using a broad range of evidence-informed modalities, including personalized nutrition and lifestyle medicine, mind-body interventions, acupuncture, herbal medicine, mistletoe therapy, loco-regional modulated electrohyperthermia (mEHT) and intravenous therapies to support symptom management and quality of life during oncological treatment (see **Figures 1**–**3**). The clinic’s shared care model operates alongside the patient’s broader medical team, with explicit positioning of not delivering alternative medicine but aiming for true integration as a key part of the cancer treatment and rehabilitation pathway.

## 2 Methods

### 2.1 Systems approach to cancer framework (clinical assessment and care planning)

Clinical assessment and care planning at Synthesis Clinic follow the Systems Approach to Cancer Framework (SACF), designed by Dr Nina Fuller-Shavel, which provides the conceptual scaffolding for the clinical work described in this paper. SACF is a four-way synthesis of (i) the six lifestyle medicine pillars (nutrition, physical activity, restorative sleep, stress management, positive social connection and avoidance of risky substances) as endorsed by the American College of Lifestyle Medicine and recently extended into oncology through the MASCC-endorsed ACLM Lifestyle Medicine Cancer Risk Reduction and Survivorship Toolkit (37); (ii) the whole-person approach framework of integrative medicine and integrative oncology, including the SIO definition of integrative oncology (13), SIO-ASCO clinical guidelines (7, 21, 22) and contemporary whole journey supportive care paradigms; (iii) the systems hubs proposed for chronic non-communicable disease (30, 38), which SACF broadens into eight clinically operational physiological domains applicable to integrative cancer care; and (iv) the contemporary hallmarks of cancer framework, including polymorphic microbiomes, non-mutational epigenetic reprogramming, and the wider Swanton “hallmarks of systemic disease” perspective (26, 27, 29).

SACF is operationalized as a practical clinical model. Each new patient consultation is structured around three integrated assessment layers. First, a detailed history is taken, covering oncological diagnosis and treatment, broader medical and surgical history, family history, current and prior medications, allergies and sensitivities, use of supplements and herbal products, environmental exposures and psychosocial context. Second, a lifestyle medicine domain review is conducted across the six pillars (combined into 5 summary hubs), identifying current strengths, modifiable factors and priorities. Third, an additional physiological systems assessment is performed across eight interlinked domains: (1) healthy microbiome balance and gastrointestinal function; (2) mental, emotional and spiritual health and biological rhythms (ultradian, circadian, infradian); (3) balanced immune function; (4) effective detoxification; (5) healthy circulation and tissue architecture (including but not limited to fascia, TME – tumor microenvironment and key processes, such as invasion, angiogenesis, metastasis and immune evasion); (6) mitochondrial and metabolic health; (7) hormone assessment across hypothalamic-pituitary-adrenal/thyroid/gonadal (HPA/HPT/HPG) and other axes; and (8) methylation, epigenetics and genomic stability. Predisposing and precipitating factors in the patient’s history that may relate to both cancer and other chronic co-morbidities and patient-defined priorities are recorded alongside this assessment.

Outputs from SACF are synthesized into a personalized Systems Approach Treatment and Care Program (SATCP) for each patient, with key input summarized graphically in **Figure 2**. The SATCP is used to prioritize management areas, sequence and select evidence-informed interventions from across the available modality panel (**Figure 3**), identify the relevant multidisciplinary team members for that patient’s current phase of care, and define a clear plan for continuing assessment and dynamic plan revision as the patient’s clinical situation and priorities evolve. The framework is therefore both a clinical assessment tool and a care planning support tool and provides the internal consistency that links the descriptive findings reported below to a coherent model of care.

### 2.2 Electronic healthcare record analysis – 2022 to 2026

Healthcare records for patients coded for their breast cancer diagnosis were analyzed from January 2022 to March 2026 inclusive as being readily available on the current electronic healthcare record system, with over 2000 consultations delivered to this patient population over the examined period. Disease stage classification combined the SNOMED-coded primary diagnosis field, the secondary diagnosis field, and the metastases location field. Patients with any of: a recorded secondary diagnosis, a recorded metastases location, or “Secondary malignant neoplasm of breast” in the primary diagnosis text were classified as ever-metastatic. All other patients with a recorded primary diagnosis were classified as primary only. Subtype classification used a hierarchical algorithm: triple positive (HR+ and HER2+) > triple negative > HER2 positive (HR- only) > HR positive (HER2- only) > unknown. Patients with no subtype flag set across all four subtype columns were classified as unknown.

Prior archival records from different clinical systems used from 2019 to 2021 were not accessed for this paper. All retrieved EHR records were filtered against opt-in electronic consent for data use for clinical audit and anonymized data publication documented on the EHR. Retrieval was done by administrative staff with analysis by study authors. Specific project approval was not required by the Synthesis Clinic internal REC (Research Ethics Committee) due to the clinical audit nature of the project.

### 2.3 Patient survey

A cross-sectional mixed-methods study was conducted from May 2025 to January 2026 in partnership with the National Centre for Integrative Oncology (NCIO; Registered Charity 1210476) and the UK Building Resilience in Breast Cancer (BRiC) Centre. Participants were recruited through three complementary channels: (i) the UK BRiC online support group network (online newsletter, members forum and partner network), reaching a community of women with current or prior breast cancer engaged with peer support; (ii) the NCIO public engagement channels, including charity newsletter, website and social media; and (iii) direct invitation of Synthesis Clinic patients with documented opt-in electronic consent for research participation, ensuring that those patients reporting on integrative oncology care had recent first-hand experience of the service. All participation was voluntary, anonymized, and informed by a study information sheet.

A bespoke 24-item online questionnaire was developed by the study team. Currently there are no established UK breast cancer questionnaires spanning both standard and integrative oncology supportive care needs. The item structure and content of our questionnaire was informed by established unmet supportive care need frameworks reported in published UK and international breast cancer research, and by the supportive care domains covered in the SIO-ASCO, ASCO and ESMO guidelines for symptom management, lifestyle support, psychological care and survivorship. The items reflecting on patient experience were structured as Likert-scale agreement statements across the relevant domains, with the same item stems used for both the standard care and IO care sections to enable direct domain-level comparison within respondents who had experienced both modes of care. Part 1 of the survey assessed demographics, cancer history, and provision of, and access to, information and support related to side effects, anxiety and fears, menopausal symptoms, sleep, nutrition and diet, exercise, psychological wellbeing, and sexual health. Psychometric analysis of Part 1 items of the survey revealed excellent reliability (Cronbach’s alpha = 0.92). Part 2 captured the same domains under integrative oncology care for the subgroup of respondents who had experience of receiving combined standard and IO care. Similarly, Part 2 items demonstrated excellent reliability (Cronbach’s alpha = 0.93).

A descriptive statistical analysis was performed, and given the descriptive aim and the asymmetric cohort sizes, no formal inferential between-group hypothesis testing was conducted. 232 breast cancer patients responded to the survey, with feedback on standard oncological care from 211 patients (the standard care cohort) and a smaller cohort of 21 patients reporting on combined standard and integrative oncology care (the IO cohort). Adherence to integrative oncology recommendations within the IO cohort was not formally assessed in this survey, as the focus was on experienced provision of, and access to, supportive care domains rather than on intervention adherence. Documenting and analyzing adherence to specific recommendations will form part of a planned prospective evaluation. To complement the survey findings, semi-structured interviews were conducted with a subgroup of breast cancer participants, with results to be reported separately in a full mixed methods analysis. Research ethics committee approval for the project was obtained (reference SYN30525).

## 3 Results

### 3.1 Baseline demographics and integrative modality use at Synthesis Clinic based on EHR data

This study combined a four-year EHR analysis of breast cancer patients attending a UK physician-led integrative oncology clinic with a cross-sectional survey of 232 women with breast cancer, comprising 211 reporting on standard oncological care alone and 21 reporting on combined standard plus integrative oncology care.

#### 3.1.1 Baseline demographics for breast cancer patients accessing Synthesis Clinic

Electronic healthcare record analysis identified 182 women with a breast cancer diagnosis who had a recorded clinic encounter at Synthesis Clinic during the period of January 2022 to March 2026, representing approximately 4.25 years of continuous service delivery specifically on this clinical system. This EHR-derived cohort is independent of the patient survey cohort reported in the survey below, as while patients will be shared across both datasets within the IO cohort, individual records cannot be matched between the two due to the anonymized nature of the survey. Therefore, the cohorts are reported separately throughout.

The full set of baseline demographics data for Synthesis Clinic breast cancer patients is reported in **Table 1**. The mean age at clinic presentation was 50.3 years (standard deviation 9.8), with a median of 51 years (interquartile range 43–57). The age distribution was skewed toward working-age women: 1.1% (n=2) were under 30, 13.7% (n=25) were aged 30–39, 28.0% (n=51) were aged 40–49, 40.7% (n=74) were aged 50–59, 14.8% (n=27) were aged 60–69 and 1.6% (n=3) were aged 70 years or older. The youngest patient was 18 years and the oldest 79 years old at presentation.

**Table 1:** Baseline characteristics of breast cancer patients attending Synthesis Clinic (EHR cohort, n=182).

Characteristic	Category	n	%
Age at presentation			
	Mean (SD), years	50.3 (9.8)	—
	Median (IQR), years	51 (43–57)	—
	<30	2	1.1%
	30-39	25	13.7%
	40-49	51	28.0%
	50-59	74	40.7%
	60-69	27	14.8%
	70+	3	1.6%
Disease stage at presentation			
	Primary only	99	54.4%
	Ever-metastatic	83	45.6%
Breast cancer subtype			
	HR positive (HER2-)	105	57.7%
	HER2 positive (HR-)	7	3.8%
	Triple positive (HR+/HER2+)	8	4.4%
	Triple negative	37	20.3%
	Unknown / not recorded	25	13.7%
Integrative oncology modality engagement			
	Nutrition consultations	170	95.0%
	Physician (integrative oncology) consultations	100	55.9%
	Intravenous nutrient therapy	38	20.9%
	Mistletoe therapy	25	13.7%
	Emotional wellbeing / EFT	24	13.2%
	Physiotherapy / rehabilitation	18	9.9%
	Herbal medicine	12	6.6%
	Yoga therapy	6	3.3%
	Oncothermia (mEHT)	4	2.2%

EHR cohort: all breast cancer patients with a recorded clinic encounter at Synthesis Clinic between January 2022 and March 2026. Disease stage derived from recorded primary and secondary SNOMED-coded diagnoses and metastases location field. Subtype assigned hierarchically: triple positive (HR+/HER2+) > triple negative > HER2+ alone > HR+ alone > unrecorded.

The EHR cohort was characterized by a substantially higher proportion of patients with metastatic disease than is seen in the general UK breast cancer population, reflecting the typical patient population referred to or self-referring to a specialist integrative oncology service. Of the 182 patients, 99 (54.4%) had primary breast cancer at presentation to the clinic and 83 (45.6%) had ever-metastatic disease (either *de novo* metastatic or primary disease that had progressed to metastatic disease at the time of clinic presentation). This metastatic representation is approximately 2.4-fold higher than that observed in the broader patient survey cohort (19.4%) and is similar to, but somewhat higher than, the ever-metastatic proportion in the integrative oncology survey cohort (42.9%).

Breast cancer subtype was assigned hierarchically using SNOMED-coded diagnoses. The cohort distribution was as follows: hormone receptor positive (HR+, HER2-) 57.7% (n=105), triple negative breast cancer (TNBC) 20.3% (n=37), triple positive (HR+/HER2+) 4.4% (n=8), HER2 positive (HR-) 3.8% (n=7), and unknown or not yet recorded 13.7% (n=25). The proportion with TNBC (20.3%) is approximately twice the population frequency of TNBC in unselected breast cancer cohorts (8–15%), consistent with the higher representation of patients with more aggressive disease in this clinical population seeking specialist support.

#### 3.1.2 Integrative oncology modality use in breast cancer patients accessing Synthesis Clinic

**Figure 4:**
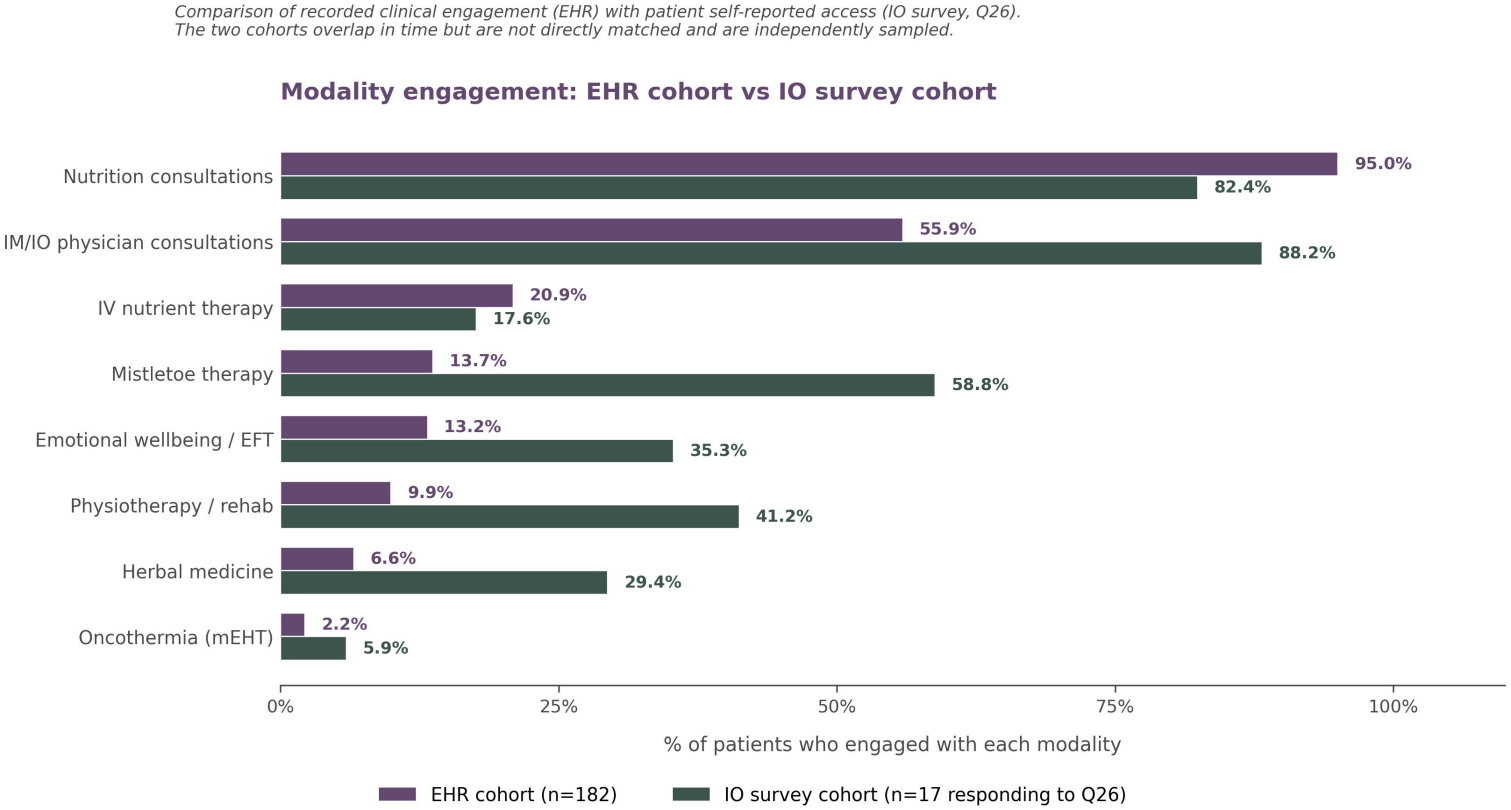
Summary of integrative oncology modality uptake by patients with breast cancer at Synthesis Clinic. This summary compares the information from EHR documentation for the whole breast cancer IO sample (n=182) with self-reported use from the smaller patient survey report (n=17 out of 21 respondents for this question). The two cohorts overlap in time but are not directly matched and are independently sampled. Yoga therapy, art therapy and health coaching are not reported above due to low engagement with these modalities in the whole cohort (<5%) and variability in access with health coaching, yoga therapy and art therapy being available at different points over 2022 to 2026.

As depicted in **Figure 4**, across the 182 patient EHR cohort, modality engagement was characterized by very high uptake of nutritional consultations and integrative oncology physician consultations, with a long tail of more specialized modalities accessed by smaller subgroups. Patients typically engaged with several modalities concurrently, consistent with a multidisciplinary care plan.

Nutritional therapy consultations were accessed by 95.0% of patients with recorded modality data, reflecting the centrality of nutritional support as the most consistently delivered element of the Synthesis Clinic care model. Integrative oncology physician consultations (separate from initial physician triage and from any nutritional or other modality consultations) were accessed by 55.9% of patients. Intravenous nutrient therapy (20.9%), mistletoe therapy (13.7%) and emotional wellbeing coaching and EFT support (13.2%) formed the second tier of accessed modalities. Physiotherapy and rehabilitation, herbal medicine, yoga therapy and oncothermia (modulated electrohyperthermia) were each accessed by between 2% and 10% of the cohort, reflecting their role as targeted modalities prescribed for specific clinical indications rather than as universally offered services, particularly with highly variable or absent reimbursement through private insurance.

### 3.2 Breast cancer patient survey results – unmet needs in standard oncological care (n=211)

Baseline characteristics of both IO and standard care cohorts and the combined breast cancer cohort of 232 patients are provided in **Table 2**. A total of 211 women with a current or prior breast cancer diagnosis provided feedback on their experience of standard oncological care alone within our survey. The cohort was predominantly aged 40–70 years (92.4%), with the largest age band 50–59 years (48.3%, n=102). Respondents were predominantly White/White British (97.2%, n=205) and well educated, with 63.1% holding a Bachelor or post-graduate degree (29.9% Bachelor, 33.2% post-graduate). Most respondents were in employment (62.6% full- or part-time), with 25.6% retired and 6.6% unable to work due to disability.

**Table 2:** Baseline demographic and clinical characteristics of UK breast cancer survey respondents (combined n = 232).

Characteristic	Category	Standard care (n=211)	Integrative oncology care (n=21)	Combined cohort (n=232)
Age (years)				
	30-39	9 (4.3%)	1 (4.8%)	10 (4.3%)
	40-49	35 (16.6%)	7 (33.3%)	42 (18.1%)
	50-59	102 (48.3%)	3 (14.3%)	105 (45.3%)
	60-69	58 (27.5%)	10 (47.6%)	68 (29.3%)
	70 and older	7 (3.3%)	0 (0.0%)	7 (3.0%)
Race / ethnicity				
	White/White British	205 (97.2%)	19 (90.5%)	224 (96.6%)
	Asian/Asian British	4 (1.9%)	1 (4.8%)	5 (2.2%)
	Another race or ethnicity (please specify)	1 (0.5%)	0 (0.0%)	1 (0.4%)
	Rather not say	1 (0.5%)	1 (4.8%)	2 (0.9%)
Highest education				
	Less than secondary school qualifications	3 (1.4%)	0 (0.0%)	3 (1.3%)
	Secondary school qualifications (GCSE, A-level, etc.)	48 (22.7%)	1 (4.8%)	49 (21.1%)
	Some university but no degree	17 (8.1%)	3 (14.3%)	20 (8.6%)
	Foundation degree	10 (4.7%)	2 (9.5%)	12 (5.2%)
	Bachelor degree	63 (29.9%)	4 (19.0%)	67 (28.9%)
	Post-graduate degree	70 (33.2%)	11 (52.4%)	81 (34.9%)
Employment status				
	Employed, working full-time	81 (38.4%)	2 (9.5%)	83 (35.8%)
	Employed, working part-time	51 (24.2%)	10 (47.6%)	61 (26.3%)
	Not employed, looking for work	2 (0.9%)	1 (4.8%)	3 (1.3%)
	Not employed, NOT looking for work	9 (4.3%)	4 (19.0%)	13 (5.6%)
	Retired	54 (25.6%)	3 (14.3%)	57 (24.6%)
	Disabled, not able to work	14 (6.6%)	1 (4.8%)	15 (6.5%)
Breast cancer subtype				
	Oestrogen receptor positive breast cancer	110 (52.1%)	13 (61.9%)	123 (53.0%)
	HER2 positive breast cancer	42 (19.9%)	3 (14.3%)	45 (19.4%)
	Triple positive breast cancer (HER2 and ER positive)	17 (8.1%)	1 (4.8%)	18 (7.8%)
	Triple negative breast cancer	20 (9.5%)	2 (9.5%)	22 (9.5%)
	Other (please specify)	22 (10.4%)	2 (9.5%)	24 (10.3%)
Breast cancer stage				
	Primary breast cancer	164 (77.7%)	12 (57.1%)	176 (75.9%)
	Secondary/metastatic breast cancer	25 (11.8%)	6 (28.6%)	31 (13.4%)
	Primary breast cancer at initial diagnosis, then secondary breast cancer	16 (7.6%)	3 (14.3%)	19 (8.2%)
	Don't know	1 (0.5%)	0 (0.0%)	1 (0.4%)
	Other (please specify)	5 (2.4%)	0 (0.0%)	5 (2.2%)

Values shown as n (%); percentages calculated against responding respondents per item. Standard care cohort (n=211): respondents reporting on standard oncological care experience, recruited through UK BRiC support group, Synthesis Clinic patients and the general public.Integrative oncology care cohort (n=21): Synthesis Clinic patients reporting on combined standard plus integrative oncology care. Combined cohort (n=232): pooled descriptive figures across both groups, presented for completeness.

Free-text "Other" subtype responses most commonly described combined ER+/HER2+ disease, lobular breast cancer, inflammatory breast cancer and DCIS.Cancer stage "Other" responses described primary cancer with regional or local recurrence.

The cohort included a clinically representative breast cancer subtype distribution: 52.1% estrogen receptor positive (ER+) breast cancer, 19.9% HER2-positive, 8.1% triple-positive (ER+/HER2+) and 9.5% triple-negative breast cancer. A further 10.4% selected “Other”. Free-text responses for this group most frequently described combined ER+/HER2+ disease, lobular breast cancer, inflammatory breast cancer and DCIS. By stage, 77.7% (n=164) reported primary breast cancer, 11.8% (n=25) had *de novo* metastatic disease and 7.6% (n=16) had primary disease followed by secondary breast cancer, giving a combined ever-metastatic subgroup of 41 (19.4%) respondents.

**Figure 5:**
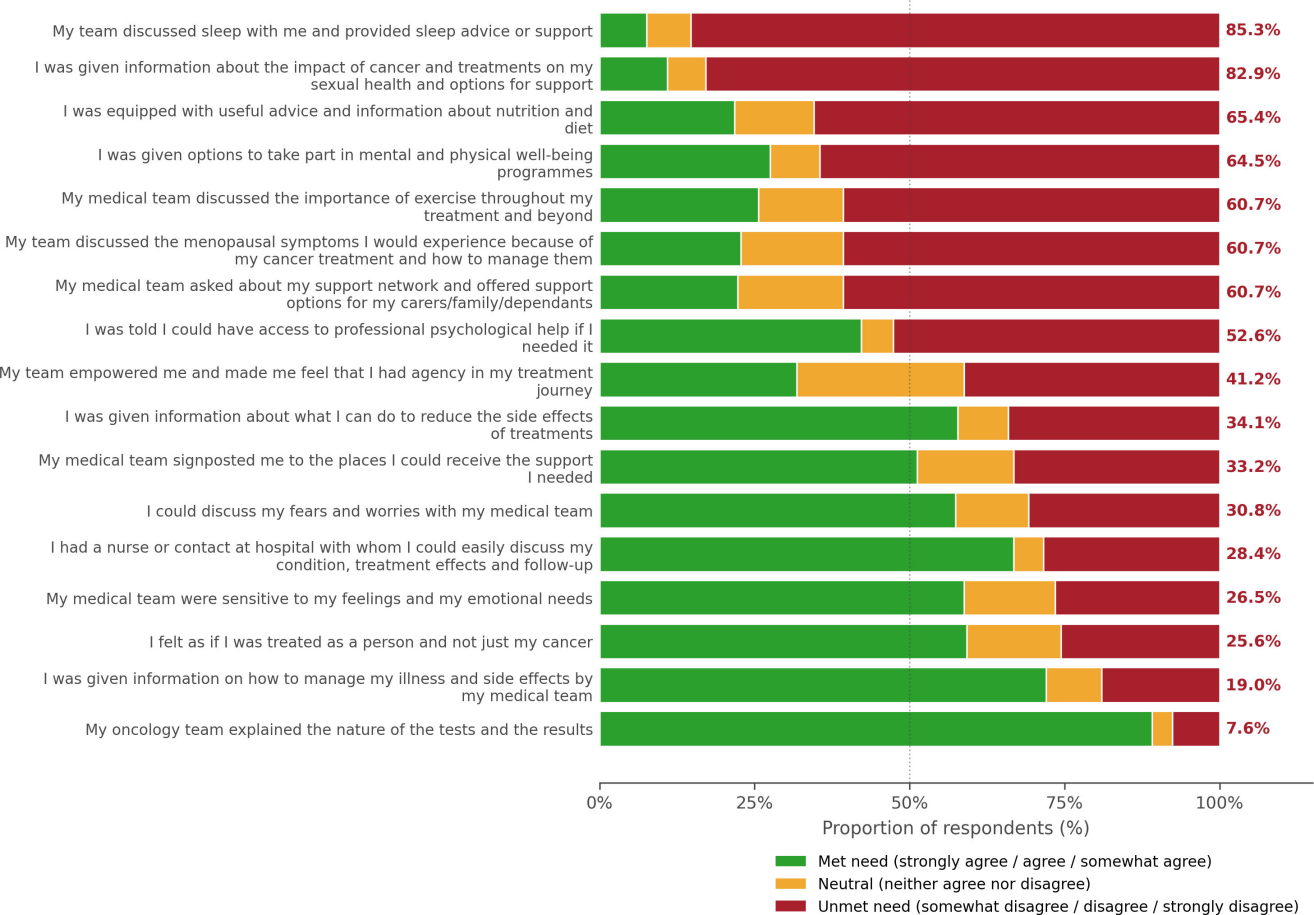
Unmet breast cancer patient needs in standard oncological care in the UK (cross-sectional survey, n = 211 breast cancer patients).

Across the seventeen domain items, two distinct patterns emerged. As **Figure 5** shows, standard care performed largely as expected on biomedical communication tasks: 89.1% of respondents agreed (strongly agree, agree or somewhat agree) that their oncology team had explained the nature of tests and results, and 72.0% agreed they had received adequate information on managing their illness and side effects. By contrast, standard oncological care support performed poorly across virtually every lifestyle, psychological and survivorship domain.

Sleep and sexual health emerged as the two most poorly addressed domains, with fewer than one in eight respondents reporting that sleep was discussed adequately (7.6% met need), and only 10.9% reporting they had received information on sexual health impact and support. Nutrition (21.8% met need), exercise (25.6% met need) and wellbeing programs (27.5% met need), which represent core lifestyle medicine domains, all fell below 30% met need. Access to professional psychological help was reported as adequately offered by only 42.2% of respondents, despite the well-documented psychological burden of breast cancer diagnosis and treatment.

Communication and relational dimensions of care performed somewhat better but still showed a substantial proportion of patients reporting unmet need: 30.8% disagreed that they could discuss their fears and worries with their medical team, 28.4% disagreed they had a hospital nurse contact for ongoing discussion, and 25.6% disagreed that they were treated as a person and not just their cancer. 41.2% disagreed that their team had empowered them and given them agency in their treatment journey.

#### 3.2.1 Patient survey results – standard care analysis of metastatic vs primary breast cancer patients

Comparing the primary disease subgroup (n=170) with the metastatic disease subgroup (n=41) revealed a broadly consistent pattern of unmet need across both groups, but with several clinically meaningful divergences. Patients with metastatic disease reported higher unmet need for nutrition advice (75.6% vs 62.9%), exercise discussion (70.7% vs 58.2%), sexual health information (90.2% vs 81.2%), and side effect reduction information (39.0% vs 32.9%). Conversely, primary disease patients reported higher unmet need around menopausal symptom management (62.9% vs 51.2%) and access to psychological help (54.7% vs 43.9%), reflecting the typical divergence in care pathways and the more medicalized follow-up structure of metastatic breast cancer care. These patterns highlight that the gap in supportive care extends into and may worsen within metastatic breast cancer populations.

### 3.3 Patient survey results – integrative oncology cohort analysis (n=21)

The integrative oncology (IO) cohort consisted of 21 Synthesis Clinic patients who completed the combined survey. Given the small IO cohort size, with n=17–21 responding per item, and the absence of a contemporaneous external comparator, the figures reported in this section are descriptive and exploratory. They should be interpreted as showing the direction and pattern of within-patient differences in reported supportive care experience between standard care and combined standard plus integrative oncology care, rather than as estimates of effect magnitude or causal effect on outcomes.

Compared with the broader 211-patient standard care cohort, the IO cohort was somewhat older (47.6% aged 60–69 years vs 27.5% in the standard care cohort), more highly educated (52.4% held a post-graduate degree vs 33.2%), and more frequently working part-time (47.6% vs 24.2%) or not in paid employment (19.0% vs 4.3%), reflecting the greater proportion of patients managing active metastatic or recurrent disease. The IO cohort had a similar distribution of breast cancer subtypes (61.9% ER- positive, 14.3% HER2-positive, 4.8% triple-positive, 9.5% triple-negative) but a substantially higher proportion with metastatic disease. 28.6% of patients had secondary/metastatic breast cancer at presentation and a further 14.3% who developed secondary disease following primary treatment, giving a combined metastatic subgroup of 42.9% (n=9) compared with 19.4% in the standard care cohort. This skew toward metastatic disease is consistent with the patient population typically referred to a specialist integrative oncology service seeking comprehensive supportive care alongside ongoing systemic therapy.

Of 17 IO cohort respondents who answered the modality question (Q26), the most commonly accessed modalities were integrative medicine consultations with a doctor (88.2%, n=15), nutrition (82.4%, n=14) and mistletoe therapy (58.8%, n=10). Physiotherapy or rehabilitation was accessed by 41.2% (n=7), emotional wellbeing coaching and EFT by 35.3% (n=6), and herbal medicine by 29.4% (n=5). IV infusion services (17.6%, n=3), art therapy (11.8%, n=2), oncothermia (mEHT, 5.9%, n=1) and health coaching (5.9%, n=1) were accessed by smaller subgroups, reflecting the personalized nature of IO care planning. Patients typically accessed several modalities concurrently within a multidisciplinary care plan as already highlighted in the EHR audit results.

#### 3.3.1 Quality and importance of integrative care in breast cancer support and closing the gap in unmet needs

Of 17 respondents answering the rating questions, 16/17 (94.1%) rated their integrative cancer care as either “very high quality” (64.7%, n=11) or “high quality” (23.5%, n=4), with one respondent rating it neither high nor low. 13/17 (76.5%) considered their integrative care “extremely important” within their overall cancer care plan, with a further 17.6% rating it “very important”, together representing 76.5% extremely important and 94.1% extremely or very important. Free-text comments explicitly described the integrative care experience as a “lifeline”, a source of “control during a time which felt out of control”, and as the place where the patient was “always … empowered and [I] have hope”.

**Figure 6:**
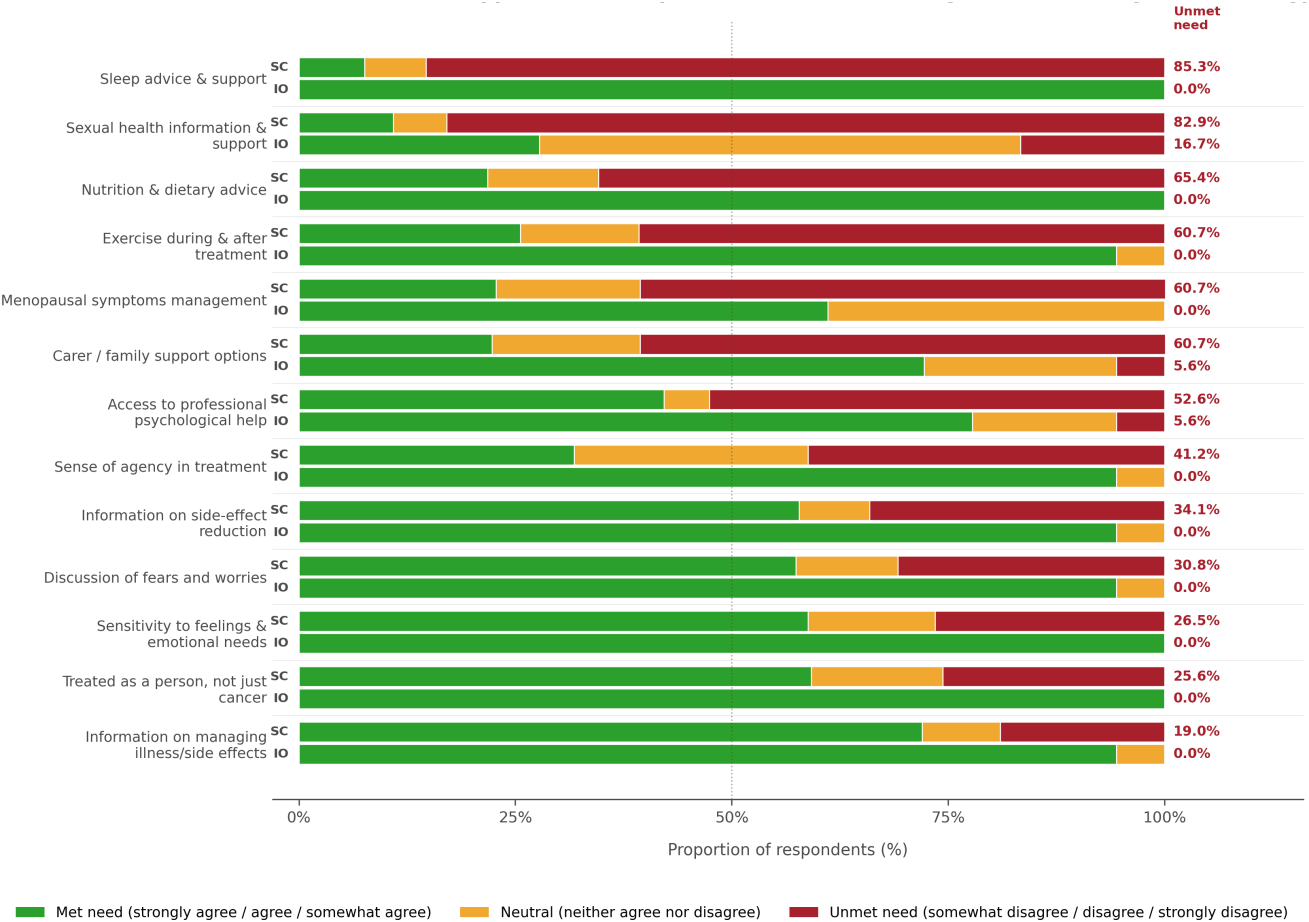
Comparison of unmet needs of breast cancer patients with standard oncological care (SC) versus integrative oncology care (IO) used alongside standard breast cancer treatment (n = 211 for standard care, n =18–21 depending on individual question response for IO care). For each domain, top bar = SC; bottom bar = IO.

As shown in **Figure 6**, across the thirteen domains for which directly comparable IO-care items were included in the survey, the IO cohort reported markedly high levels of met need. 100% met need was reported for sleep advice and support, nutrition and dietary advice, person-centered approach, and sensitivity to feelings and emotional needs. Met need exceeded 94% for information on managing illness and side effects, information on the management of side effects, discussion of fears and worries, exercise discussion, and empowerment/agency in treatment. Menopausal symptom management showed 61.1% met need with 38.9% selecting “neither agree nor disagree”, likely reflecting respondents who had not yet experienced these symptoms or were post-menopausal at diagnosis. Sexual health information and support remained the lowest-performing domain even within IO care (27.8% met need, 55.6% neutral, 16.7% unmet).

When the same patients reported on their experience of both standard and integrative oncology care, unmet need was strikingly eliminated or near-eliminated across all thirteen domains examined. Across all thirteen domains examined, IO care reduced unmet need; in 11/13 domains unmet need was reduced to 0%. The largest absolute reductions were seen in nutrition (−91 percentage points), sleep (−86 pp), and exercise (−71 pp), which are the three core lifestyle medicine domains integral to high quality breast cancer care and covered in ASCO and ESMO guidelines for people impacted by cancer (39, 40). Patient-reported experience of being “treated as a person and not just my cancer” moved from 38.1% unmet to 100% met need within the same individuals.

One key domain retains measurable residual unmet need even within the IO cohort. Sexual health information and support (16.7% unmet, 55.6% neutral, 27.8% met) was less consistently addressed. As Synthesis Clinic does not currently have its own psychosexual support service, this is flagged for explicit incorporation into future service development to improve breadth of coverage and service quality.

## 4 Discussion

The findings reported here can be read as a real-world description of a UK integrative oncology service operating under the Systems Approach to Cancer Framework. The domains in which standard UK breast cancer care is most consistently underserving patients, including sleep, nutrition, exercise, mental and physical wellbeing, sexual health and menopausal symptoms, map directly onto the lifestyle medicine pillars and the systems hubs that SACF is designed to address, and onto the supportive care domains that the SIO-ASCO, ASCO, MASCC and ESMO guidelines and publications explicitly cover. The convergence of these three layers (observed unmet need, framework structure and guideline alignment) provides the internal logic for the rest of this Discussion focused on the gap between routine UK breast cancer care and contemporary evidence-based supportive and integrative care being both sizeable and amenable to a systems-level multidisciplinary response.

### 4.1 Key findings

Three principal findings emerged. First, the experience of standard UK oncological care for breast cancer is bifurcated. Biomedical communication tasks are handled adequately for most respondents, but lifestyle, psychological, sexual health, menopausal and survivorship support are systematically and severely under-delivered, with majority unmet need across seven supportive care domains. Second, the domains in which standard care most consistently fails are those for which both standard care and integrative oncology have accumulating guidelines in place (7, 21, 22, 39, 40), and within our IO cohort the corresponding unmet need was reduced to 0% in 11 of 13 directly comparable domains. Third, the qualitative free-text responses from both cohorts converge on a coherent narrative of patients seeking, and benefiting from, structured holistic care that they could not otherwise access through standard NHS oncology pathways. These findings will be combined with interview data in a full mixed methods analysis in due course.

### 4.2 Integrative modality use in patients with breast cancer attending a UK integrative oncology clinic

The Synthesis Clinic EHR cohort comprises 182 women with breast cancer attending the clinic between January 2022 and March 2026. The cohort is enriched for patients with metastatic disease (45.6% ever-metastatic) and for triple-negative biology (20.3%), reflecting the typical referral and self-referral pattern to a specialist integrative oncology service. Patients access a structured panel of evidence-informed modalities, predominantly anchored on nutritional therapy and integrative oncology physician consultations, with a minority accessing additional specialized modalities (mistletoe therapy, IV nutrient therapy, emotional wellbeing support, herbal medicine, physiotherapy, yoga therapy and oncothermia) selected on the basis of individual clinical need and accessibility.

EHR modality engagement patterns align broadly with survey self-reports, but the integrative oncology survey cohort reported substantially higher rates of access to most modalities (e.g., 88.2% vs 55.9% for physician consultations; 58.8% vs 13.7% for mistletoe therapy; 41.2% vs 9.9% for physiotherapy) than was recorded across the broader EHR population.

Two considerations help interpret these differences. First, the IO survey cohort represents a smaller and more engaged sample of patients who self-selected to complete a detailed survey of their integrative oncology experience, and this subpopulation is therefore likely to be over-representative of patients who accessed multiple modalities. Second, the EHR field captures only modality encounters that have been explicitly recorded as a separate clinical episode within the structured EHR. This may undercount engagement with broader physician-led activities (such as embedded mistletoe prescription within a routine integrative oncology consultation) that are clinically delivered but have not been consistently separately coded. Yoga therapy, art therapy and health coaching are not reported in the comparison figure due to low whole-cohort engagement (<5%) and variability in access, with these modalities offered at different points across the 2022 to 2026 service period.

#### 4.2.1 Equity and representation considerations in integrative oncology data

A consistent and important limitation across all three components of this work is the marked skew of the participating cohorts toward White British, well-educated and economically active women. In the standard care survey cohort (n=211), 97.2% identified as White or White British and 63.1% held a Bachelor’s or post-graduate degree (33.2% post-graduate). In the IO survey cohort (n=21), this skew was even more pronounced: 90.5% White or White British and 86% educated to degree level, with 52.4% holding a post-graduate qualification. The Synthesis Clinic EHR cohort (n=182) draws predominantly from a private fee-paying patient base and is therefore inherently selected toward those with the financial means (especially in the face of highly variable insurance coverage for integrative care), English language fluency, health literacy and to an extent, geographic proximity to access a specialist integrative oncology service in southern England. These patterns mirror the well-documented inequities in complementary and integrative medicine use among UK and international cancer populations, where ethnic minority groups, lower-socioeconomic-status patients and those for whom English is not the first language are systematically under-represented despite comparable or higher symptom burden and supportive care need (41–43). The findings reported here therefore should not be read as generalizable to UK breast cancer patients, and the encouraging results from the IO cohort should be interpreted with the explicit caveat that they describe the experience of a structurally privileged subgroup. Addressing this equity gap is central to translating the model described here into a pathway accessible to all UK breast cancer patients, and this is the explicit focus of the UK National Centre for Integrative Oncology (NCIO), where equitable access to integrative oncology support and education for both patients and healthcare professionals is a core part of its charitable mission.

### 4.3 Unmet supportive care needs in standard breast cancer care

Our standard care findings are consistent with, and in several domains more pronounced than, the international literature on unmet needs in breast cancer survivors. A 2023 systematic scoping review of 77 studies identified social support (74%), daily activity (54%), sexual or intimacy concerns (52%), fear of recurrence (50%) and information needs (45%) as the most common unmet supportive care domains in BC survivors (44). An Australian systematic review of 35 studies reported point-prevalence unmet need of up to 38% for fear of cancer spreading in BC patients (45), and a UK survey of 143 women living with metastatic BC (LIMBER) found that only 56% had access to a specialist nurse and only 51% had been offered any additional support (46). Sexual health needs in BC survivors are particularly poorly addressed internationally. A recent Portuguese mixed-methods study in 336 survivors documented critical unmet sexual health needs alongside low sexual functioning and high rates of pain and emotional distress (47), and a UK scoping review highlighted the consistent under-recognition of sexual and informational concerns in routine oncology (48). Our finding of 83% unmet need for sexual health information and support within standard of care sits at the upper end of this international evidence base.

The 85% unmet need for sleep advice and 65% for nutrition observed in our cohort similarly matches and extends prior literature. Symptom-cluster analyses identify fatigue–sleep disturbance as one of the two most commonly reported clusters in BC patients across the treatment trajectory, yet a recent Asia-Pacific multinational survey found existential survivorship and comprehensive cancer care to be the highest unmet domains across every studied country, with sleep disturbance affecting a majority of cancer survivors and physical symptom concerns largely going unaddressed (49). Our subgroup analysis showed that ever-metastatic patients reported particularly high unmet need for nutrition (76%), exercise (71%) and sexual health (90%) information, broadly consistent with the LIMBER finding that women with metastatic BC describe an inconsistent and inadequate level of supportive care provision (46), and with international evidence that supportive care service utilization among long-term metastatic BC patients remains low even in well-resourced cancer centers (50). The young women’s literature highlights an additional layer of complexity. Unmet information needs predict subsequent anxiety in early survivorship, and 41% of young women report elevated anxiety in early survivorship alongside high unmet psychological needs (51). The substantive unmet psychological needs reported by our standard BC cohort highlight the urgency to address anxiety and depression through consistent application of guideline-based care (21, 52) with prompt intervention to prevent escalation into survivorship. The increased risk for anxiety, depression and suicide in BC is well documented (53). A large recent meta-analysis found that anxiety and depression increase the mortality risk by up to 30%, with factors such as the lack of social support and younger age moderating this risk (54). Both contribute substantially to poor rates of medication and treatment adherence (55). Around one-third of BC patients initially diagnosed with post-traumatic stress disorder (PTSD) continue to experience similar or worsened PTSD after four years (56), and the risk of suicide in women with a BC diagnosis exceeds that of the general population, and is the greatest among female-specific cancers (57). It is therefore imperative that evidence-based psychological support is an integral component of the cancer care pathway in the UK and goes beyond the current over-reliance on counseling offered via charities.

Two contextual observations from the qualitative data warrant emphasis. First, 17% of substantive free-text responses identified UK charities (most commonly Maggie’s, Macmillan, BRiC and local cancer centers) rather than NHS oncology teams as the actual source of holistic support but it is essential that these institutions and emerging support organizations, such as the NCIO, receive sustainable funding if they are to deliver core components of supportive care. Second, 18% of substantive responses described mental health distress that emerged or worsened after the end of active treatment, in keeping with longitudinal evidence that emotional distress and unmet psychological need persist well into long-term survivorship (58, 59) and that survivors face an ongoing search for a ‘new normal’ alongside identity loss, isolation and uncertainty (59).

### 4.4 Integrative oncology as a guideline-aligned response to close the unmet need gap

The aims of integrative oncology as a patient-centered, evidence-informed field of comprehensive cancer care are to optimize health, quality of life and clinical outcomes (13). Crucially, the seven domains of majority unmet need identified in the broader UK survey - sleep, sexual health, nutrition, mental and physical wellbeing programs, exercise, menopausal symptoms and carer support - map almost directly onto the topics covered by recent and planned SIO-ASCO guidelines.

ASCO endorsed the 2018 SIO clinical practice guideline on integrative therapies during and after BC treatment, providing graded recommendations for music therapy, meditation, stress management and yoga (anxiety/stress); meditation, relaxation, yoga, massage and music therapy (depression/mood); meditation and yoga (quality of life); and acupressure and acupuncture (chemotherapy-induced nausea and vomiting) (23). The 2022 SIO-ASCO pain management guideline recommended acupuncture for aromatase-inhibitor-related joint pain, which is directly relevant to the long endocrine therapy duration described in our cohort, and acupuncture, reflexology or acupressure for general cancer or musculoskeletal pain, hypnosis for procedural pain and massage during palliative or hospice care (22). The 2023 SIO–ASCO guideline on anxiety and depression in cancer recommended mindfulness-based interventions, yoga, relaxation, music therapy, reflexology and aromatherapy during active treatment, with mindfulness, yoga, acupuncture, tai chi/qigong and reflexology post active treatment (21). The 2024 ASCO–SIO update on cancer-related fatigue recommended exercise, cognitive behavioral therapy, mindfulness-based programs and tai chi/qigong during and after treatment, with American ginseng during treatment and yoga, acupressure and moxibustion after treatment (7). In parallel, the ASCO 2023 anxiety and depression survivorship guideline endorses a stepped-care model of psychological, educational and psychosocial interventions (52), and the ASCO nutrition and physical activity guideline provides diet, physical activity and weight recommendations directly applicable to BC patients (39).

The evidence base supporting structured lifestyle interventions has matured rapidly. Two large meta-analyses encompassing several hundred thousand patients show that meeting post-diagnosis physical activity guidelines is associated with approximately a 30-40% reduction in all-cause mortality and a 30% reduction in BC-specific mortality, with dose-response relationships extending well beyond the 150 minutes-per-week threshold (60, 61). A 2025 meta-analysis of 50,689 BC survivors confirmed that meeting aerobic exercise guidelines is associated with an approximately 50% reduction in the hazard ratio for all-cause mortality (62). Physical activity also improves health-related quality of life across breast cancer-specific domains (63), and structured exercise has been shown to improve aromatase-inhibitor-related arthralgia and adherence to endocrine therapy (64). This is particularly relevant given the pooled five-year endocrine therapy adherence rate of approximately 66% and the consistent finding that side effect burden and depression are key drivers of non-adherence (65). Yet, despite this evidence and clear guideline endorsement, our 211-patient cohort reported 61% unmet need for exercise advice and 65% for nutritional advice rising to 71% and 76% respectively in the metastatic subgroup. The gap between guideline recommendation and routine UK delivery is therefore substantial and must be consistently addressed.

Within our IO cohort, mistletoe (*Viscum album* extract; VAE) therapy was the third most accessed modality, used by 59% of respondents alongside integrative medicine consultations (88%) and nutrition (82%). Mistletoe therapy is widely used and reimbursed as adjunctive supportive care in continental European integrative oncology to improve quality of life during cancer treatment (36, 66) and has accumulating evidence for improving cancer-related fatigue, with a 2022 systematic review and meta-analysis reporting a moderate effect size in randomized controlled trials (standardized mean difference −0.48) that is comparable to physical activity (67).

Complementary medicine use among BC patients worldwide is high and largely independent of clinician oversight. This widespread, often undisclosed, patient-initiated use carries genuine clinical risk, including herb/supplement-related interactions. An integrative review and clinical practice guideline by Balneaves and colleagues, and joint ASCO–SIO statements have therefore explicitly recommended that oncology services proactively assess complementary medicine use, document it in the medical record, and provide structured decision support rather than allowing the field to remain a parallel patient-driven activity (68).

The Synthesis Clinic model is consistent with this guidance. The clinic operates as a CQC-registered, physician-led, multidisciplinary outpatient service in which IO physicians, registered nurses and nurse prescribers, BANT-registered nutritional therapy practitioners and dietitians, physiotherapists, clinical psychology and emotional wellbeing coaching, yoga therapy and licensed acupuncture and herbal medicine practitioners work together, supported and guided through four multidisciplinary team meetings per week under explicit clinical governance, including a clinical audit and patient reported outcome measures program. All supplement and herbal prescriptions are screened for drug-supplement and herb-drug interactions by both clinicians and an integrative clinical pharmacist, with additional safety monitoring for higher-complexity modalities, such as mistletoe therapy. This structured governance and personalized risk-benefit assessment, alongside robust information gathering through the EHR and shared care arrangements with partner oncology providers, is the mechanism by which integrative oncology can be delivered safely and reliably alongside standard treatment.

### 4.5 Scalability and transferability within the NHS

The Synthesis Clinic operates as a CQC-registered private outpatient service in southern England, with clear implications for scalability and equity. Context-specific elements include the private care model, the availability of intravenous nutrient and mistletoe therapies in an outpatient setting, and the geographic reach of a single regional center. Elements potentially transferable to publicly funded UK cancer pathways include the Systems Approach to Cancer Framework as a clinical assessment scaffold working alongside the HNA (Holistic Needs Assessment), the SATCP as a personalized care planning tool, the structured PROMs panel (PROMIS Global Health, MDASI, and WEMWBS) for ongoing service evaluation, integrative clinical pharmacy screening of supplement and herbal medicine interactions, multidisciplinary integrative care team meetings, and shared care positioning alongside oncology providers. Nurse and allied health professional-led models, as outlined in the precision health vision for advanced practice cancer nurses (69) and the MASCC-endorsed lifestyle medicine toolkit (37), offer a practical staffing template for adaptation within NHS settings.

Our considered position is that multidisciplinary integrative oncology hubs of the type described here should not be retrofitted into single primary care or single secondary care settings, where they may be diluted by competing demand and where the necessary multidisciplinary, governance, and PROMs infrastructure is hard to sustain. Instead, they could be best developed as specialist community-based centers operating within the NHS pathway, working in shared care with both primary and secondary care teams. This position is directly aligned with the National Cancer Plan for England 2026. The Plan’s central ambitions, particularly in driving up quality of life for people living with cancer by shifting more care into communities to deliver personalized support throughout and beyond treatment, all require a model of supportive and integrative care that sits between the GP and the acute hospital and that is sized for the complexity of multimorbid, long-term cancer care. Specific commitments in the Plan reinforce this. Every patient is to receive a personalized assessment of their needs and a personal cancer plan covering physical, mental health and social needs (the natural domain of an SATCF/SATCP-style assessment), and Action 15 explicitly incentivizes the shift of cancer care into a smaller number of specialist centers on the basis that outcomes are better when patients are treated by teams with concentrated expertise. We propose that specialist integrative oncology community hubs, embedded within cancer alliances and the Neighbourhood Health Service, are the most clinically and operationally coherent vehicle to deliver on the Plan’s living-well-with-cancer ambition for the supportive care domains where routine care currently underperforms.

Significant barriers to implementation remain, including the absence of NHS commissioning routes for multidisciplinary integrative oncology services, the limited UK availability of formally trained integrative oncology physicians, nurses, AHPs and clinical pharmacists in the UK, the lack of nationally agreed competency frameworks for IO providers, and the need for a UK-relevant evidence base on cost, outcomes and equity of access. Addressing these barriers is a central focus of the UK National Centre for Integrative Oncology (NCIO) and aligns directly with the Plan’s emphasis on workforce development, quality standards, multi-year budgets that enable investment in long-term outcomes, and quality improvement collaboratives convened through cancer alliances.

### 4.6 Strengths and limitations

This EHR analysis has several features that distinguish it from the broader UK breast cancer real-world evidence base. To our knowledge, it is the first published UK real-world cohort describing integrative oncology service delivery and modality engagement in breast cancer. Although the UK has well-established conventional oncology real-world data infrastructure, none of these sources captures structured IO delivery, and NHS multimodal rehabilitation services such as the Sheffield Active Together program (70) cover prehabilitation and rehabilitation but not the broader IO support described here. The closest international comparators - the German Havelhöhe breast cancer center cohort (n=231), which evaluated patient-reported internal coherence and resilience associated with non-pharmacological interventions (71), and a multicenter Italian retrospective study (n=54) reporting feasibility and tolerability (72) - are smaller or focused on different outcomes, so the Synthesis Clinic cohort (n=182) contributes a new and complementary perspective and addresses an explicit UK evidence gap. The cohort is substantial relative to comparable IO publications and large enough to support meaningful description of nine modalities. The analysis draws on SNOMED-coded structured EHR fields, allowing rigorous classification of disease stage and subtype rather than reliance on self-report or unstructured free-text. The high ever-metastatic proportion (45.6%, vs 19.4% in the survey cohort) demonstrates that the IO service is reaching the patients most affected by ongoing supportive care need. The consistency between EHR-derived engagement and the parallel survey supports the validity of both data streams. Its limitations include modality engagement recorded as a binary engaged/not-engaged flag rather than intensity of engagement (a longitudinal analysis of modality use and concordance with care pathway is planned), unrecorded breast cancer subtype in 13.7% of patients (incomplete legacy records or pending diagnoses, with data completion ongoing), and derivation from a single specialist, physician-led multidisciplinary center, limiting representativeness and precluding comparison with single-modality, non-medically-guided support.

The patient survey component has complementary strengths and limitations. Its principal strengths are a standard-care sample (n=211) comparable to or larger than several published international BC unmet needs surveys, a directly comparable IO cohort with paired Likert items enabling within-patient analysis, a multi-source design combining survey data with EHR-derived service description, and explicit alignment between survey domains and the published guidelines across on-treatment support and survivorship care. The limitations are equally clear. The survey was cross-sectional and self-administered, recruited principally through the UK BRiC support group, the general public, and Synthesis Clinic patients, with consequent potential selection toward more engaged and better-educated respondents. The IO cohort (n=21) is small, drawn from a single specialist clinic and inherently self-selected, making the within-patient comparison descriptive rather than causal and leaving recall bias regarding the standard-care experience unexcluded. Some residual unmet need persisted within IO care, particularly in sexual health, reflecting an evolving service that ongoing quality improvement will need to address.

Across both data streams, the dominant shared limitation is the sociodemographic skew detailed in Section 4.2.1. The findings describe a structurally privileged subgroup and are not generalizable to all UK breast cancer patients. Wider sociodemographic representation is being pursued through the NCIO outreach program and planned broader sampling, and a full mixed-methods analysis combining the survey with semi-structured patient interviews is in preparation, which will provide a more nuanced thematic understanding of the experiential differences between standard and integrative care.

### 4.7 Future directions

Within-patient comparison in our study demonstrates that the same individuals who reported very high unmet need under standard care reported near-uniform met need under IO care, with reductions in unmet need exceeding 50 percentage points in 8 of 13 domains and reaching 0% unmet need in 11 of 13 domains. These figures are descriptive within-patient comparisons in a small (n=21), self-selected IO cohort and should be interpreted as a directional signal rather than as an estimate of effect size or a test of intervention effectiveness. The study was not designed to evaluate efficacy and provides no controlled comparison between standard and integrative oncology care. The interpretation is accordingly not that integrative oncology is the only, or even the principal, answer to unmet supportive care need in BC. It is, rather, that a structured, multidisciplinary, physician-led SACF model of holistic support - explicitly addressing sleep, nutrition, exercise, psychological support, sexual health, menopausal symptoms and carer support, with safety governance for any complementary modalities used - is feasible, and is rated by the patients receiving it as both highly important (76% extremely important) and high quality (88% very high or high). This converges with patient-led calls for re-imagined metastatic BC care emphasizing holistic support, referrals to non-oncology services, acceptance of integrative medicine and personalized long-term planning (73) with the optimal supportive care framework by Kida and colleagues - personalized, interdisciplinary care combining symptom management, psychosocial support, physical activity, nutrition and advance care planning by disease phase (74). The Synthesis Clinic model represents one practical implementation of this framework within the UK healthcare ecosystem.

Building on this model, the National Centre for Integrative Oncology (NCIO; Registered Charity 1210476, www.ncio.org.uk) was established to address two interlinked gaps these analyses make clearly visible. Firstly, despite substantial international evidence and the SIO-ASCO guideline recommendations, evidence-informed integrative oncology care remains inaccessible to the majority of UK cancer patients within the NHS pathway, with highly limited provision through a single London NHS center. Secondly, the limited UK private integrative oncology services that do exist are overwhelmingly accessed by patients from White British, well-educated and economically active backgrounds, leaving people most affected by unmet supportive care need - minority ethnic communities, lower-socioeconomic-status backgrounds, and people facing language, geographic or financial barriers - without awareness of, or meaningful access to, IO as a supportive intervention. NCIO is building a medically-led multidisciplinary integrative oncology service for adults, teens and children impacted by cancer, and their families and carers, with the explicit aim of equitable access to evidence-informed IO support across the UK. Its work spans three complementary streams: (i) direct patient and carer support via free telehealth consultations, helpline support, and a dedicated app developed in partnership with the Oncio platform, prioritizing under-served populations; (ii) educational programs for patients, families and carers, to build health literacy and informed decision-making around integrative options, alongside continuing professional development programs for oncology, primary care and allied health professionals; and (iii) research, including the Growth and Resilience in Trauma (GRiT) Centre, supporting the development and implementation of integrative oncology programs alongside standard NHS care. The present findings directly shape NCIO’s strategic priorities, particularly the need for accessible, language-inclusive supportive care content addressing sleep, nutrition, exercise, sexual health, menopause and psychological well-being, which are the domains in which UK standard care is most consistently failing to meet patient need.

Several extensions projects stemming from this work are already in progress. The Synthesis Clinic patient-reported outcome measures program (PROMIS Global Health, MDASI, MSQ and WEMWBS) is being collated across breast and gynecological cancers, and the full mixed-methods analysis of the breast cancer patient survey and accompanying interview data, building on initial poster presentation at ESMO 2025 (10), is in preparation. Further work is needed to define optimal service models, costings, equity of access, and longer-term outcomes including patient-reported quality of life, treatment adherence, and survival. The magnitude and consistency of unmet supportive care needs documented here, set against the broader evidence base, justifies greater research investment in the UK multidisciplinary integrative oncology service delivery.

The present study is not designed to evaluate the efficacy of integrative oncology and should not be read as effectiveness evidence. It does, however, describe a feasible, structured, multidisciplinary delivery model and a within-patient pattern of reduced unmet supportive care need that, taken together with the wider international evidence base on lifestyle medicine, mind-body interventions, and structured supportive care, supports a prospective controlled evaluation of multidisciplinary integrative oncology hubs within the NHS. England’s 2026 National Cancer Plan set an ambitious goal: by 2035, 3 in 4 people diagnosed with cancer will be cancer-free or living well at 5 years. It commits to a community shift in cancer care, personalized cancer plans, named neighborhood care leads, national digital-first prehabilitation by 2028, and modernized multidisciplinary team working. Delivering on this living-well-with-cancer ambition for breast cancer survivors will require systematic investment in equitable, multidisciplinary, person-centered supportive and integrative care - care that closes the unmet need gaps documented here, delivered by trained teams in specialist community hubs working in shared care with primary care and hospital cancer services. 

## Data Availability

The original contributions presented in the study are included in the article/supplementary material. Further inquiries can be directed to the corresponding author.
